# EPHB1 Protein Promoted the Progression of Prostate Adenocarcinoma Through Phosphorylating GSK3B and Activating EPHB1-GSK3B-SMAD3 Pathway

**DOI:** 10.1155/humu/4961883

**Published:** 2025-06-12

**Authors:** Bohan Xu, Shen Lin, Kai Yang

**Affiliations:** ^1^Department of Urology, The First Affiliated Hospital, Zhejiang University School of Medicine, Hangzhou, China; ^2^Cancer Center, Zhejiang University, Hangzhou, China; ^3^Department of Urology, Center for Reproductive Medicine, the Fourth Affiliated Hospital of School of Medicine, and International School of Medicine, International Institutes of Medicine, Zhejiang University, Yiwu, China

**Keywords:** apoptotic characteristics, cell coculture, co-immunoprecipitation, prostate adenocarcinoma, western blot

## Abstract

**Background:** The apoptosis affected the prostate adenocarcinoma (PRAD); we aimed to explore the potential pathogenesis of high-risk patients based on the apoptosis features.

**Method:** The RNA-seq data of patients and apoptosis genes were used for apoptosis score calculation via “GSVA” package; then, the weighted gene coexpression network analysis (WGCNA) and Lasso algorithm were performed for a RiskScore model. After that, the “maftools” package was applied for the somatic mutation analysis. By combining the Kaplan–Meier (KM) survival curves in order to compare the prognosis of different subgroups of patients, Cell Counting Kit-8 (CCK-8), EdU staining, and Transwell assays were performed. Protein expression was measured using western blotting. Finally, the activity of PRAD cells in macrophage polarization was detected using coculture and immunofluorescence assays.

**Results:** The PRAD samples had significantly lower apoptosis scores, and the RiskScore supported the risk stratification of patients. In somatic mutation analysis, *EPHB1* and *KIF13A* from the top six mutant genes were overexpressed in 22RV1 and PC-3 tumor cells, and low levels of *EPHB1* indicated a better prognosis. Overexpression or knockdown of *EPHB1* affected cell viability, proliferation, and invasion. We found that high expression of *EPHB1* interacting with GSK3B protein promoted the expression of *p-SMAD3* in 22RV1 cells with high levels of antiapoptotic and invasion markers (*BCL2*, *Snail*, and *N-CAD*). Importantly, *GSK3B* and *EPHB1* knockdown inhibited *p-SMAD3* activation and promoted proapoptotic features, accompanied by a reduction in macrophage M2 polarization.

**Conclusion:** This study revealed that *EPHB1* plays a pivotal role in activating the EPHB1-GSK3B-SMAD3 pathway to facilitate PRAD progression.

## 1. Introduction

Prostate adenocarcinoma (PRAD) is a common male malignancy with an increasing incidence rate as the population ages [1] and is clinically considered a strongly localized neoplasm with slow progression. The pathogenesis of PRAD remains poorly understood [2]. Epidemiological studies have revealed that obesity, smoking, alcohol use, and genetic factors are associated with a high risk of PRAD [3]. Improved diagnostic and treatment techniques have increased the 5-year overall survival (OS) by up to 90%; however, distant metastasis often occurs in advanced PRAD with invasive features and lowers the 5-year OS rate to 30% [4]. Although radical prostatectomy, radiation therapy (RT), and androgen deprivation therapy (ADT) contribute to favorable treatment outcomes [5], existing diagnostic techniques lack the specificity of distinguishing the aggressive and nonaggressive properties of PRAD, often resulting in treatment failure [6]. Some patients with highly malignant PRAD are insensitive to ADT and have an OS that is 80% similar to that of patients who do not receive ADT [7]. Castration-resistant prostate cancer (CRPC) is resistant to both chemotherapy and radiotherapy [4]. To date, clinicians have relied on clinicopathologic characteristics—such as prostate-specific antigen levels, TNM staging, and Gleason score—to guide therapeutic decisions and assess prognosis in patients with PRAD [8]. However, these markers have shown limited precision in clinical practice. Therefore, it is necessary to develop a novel and effective risk model that integrates molecular profiles with clinical data [9].

Apoptosis is a form of programmed cell death that mediates the efficient removal of damaged or harmful cells produced by oxidative stress, genotoxic stress, or hypoxia [10]. Caspases (aspartate-specific proteases) are the core mechanisms of apoptosis and regulate the imbalance between antiapoptotic (*Bcl-xL*, *Bcl-2*, *Mcl-1*, *A1/Bfl-1*, and *Bcl-w*) and proapoptotic (*Bax, Bak*, and *Bok/Mtd*) regulators of homeostasis and carcinogenesis [11]. For example, inactivation of the tumor suppressor P53 causes carcinogenesis by reducing apoptosis of tumor cells [12], and targeting apoptotic molecules could be developed as a treatment strategy to overcome tumor apoptotic resistance [13]. For apoptosis research in PRAD, Raffo et al. demonstrated that overexpression of antiapoptotic *Bcl-2* protects PRAD cells from apoptosis [14] and that overexpressed antiapoptotic proteins *Bcl-X* and *Mcl-1* in high-grade and metastatic PRAD promote cancer resistance to apoptosis [15]. Interferon activates caspase-8 to increase tumor responsiveness to chemotherapy-induced apoptosis [16], and inducing the P53 gene enhances the radiosensitivity of PRAD to P53 gene therapy [17]. These findings suggest that apoptotic pathways and processes play crucial roles in PRAD progression. In addition, other apoptotic pathways include tumor necrosis factor receptor (TNFR) systems of TNFR1-TNF*α*, TRAILR2 (DR5)-TRAIL, TRAILR2 (DR5)-TRAIL, and FAS (CD95, APO-1)-FasL [18], especially tyrosine/serine receptor kinases of the EPH receptor B1 (EPHB1). Studies have found that EPHB1 is upregulated by activating PI3K/Akt/NF-*κ*B signaling in a chronic ocular hypertension (COH) model used to analyze the apoptosis of retinal ganglion cells (RGCs) [19] and that EPHB1 is also involved in the regulation of apoptosis during PRAD progression [20]. These findings suggest that exploring apoptotic features is promising for understanding the potential pathogenesis of PRAD and developing effective therapeutic targets. Here, we conducted a retrospective study in a PRAD cohort collected from The Cancer Genome Atlas (TCGA), based on apoptosis-related genes. PRAD samples had significantly higher apoptosis scores, and a risk score model for risk stratification in PRAD was constructed by applying the weighted gene coexpression network analysis (WGCNA) and Lasso algorithms. Focusing on the top six mutant genes in high-risk samples, an in vitro assay demonstrated that *EPHB1* and *KIF13A* were significantly overexpressed in tumor cells and that low levels of *EPHB1* were associated with a better prognosis. The role of *EPHB1* in tumor viability, proliferation, and invasion was analyzed using knockdown and overexpression experiments. Furthermore, the role of *EPHB1* protein in the phosphorylation of Glycogen Synthase Kinase 3 Beta (GSK3B) and SMAD Family Member (SMAD3) proteins, as well as the effects of apoptotic proteins, was examined by western blotting. Overall, the aim of this study was to reveal the critical role of EPHB1 in PRAD progression, and it was found that it activates SMAD3 phosphorylation through interactions with GSK3B to promote tumor cell survival, invasion, and the formation of an immunosuppressive microenvironment, which provides new evidence and perspectives on the progression mechanism of PRAD.

## 2. Materials and Methods

### 2.1. Cell Culture and Gene Expression Testing

Human normal prostate epithelial cell line (RWPE-2) and human prostate cancer cell lines (22RV1, PC-3, C4-2, C4-2B, and DU145) were obtained from the American Type Culture Collection (ATCC). Dulbecco's Modified Eagle's Medium (DMEM) (OMDCM025, Oumashi, Shanghai, China) supplemented with 10% fetal bovine serum (FBS) (Gibco, Waltham, Massachusetts, United States) and 1% penicillin/streptomycin (P/S, Gibco, United States) was used for RWPE-2 cell culture. The RPMI-1640 medium (Gibco, United States) containing 10% FBS and 1% P/S was used to culture the 22RV1 and PC-3 cells. All cells were maintained in incubator with 5% CO_2_ at 37°C. STR identification has been carried out on the cells, and the outcome of mycoplasma detection shows negativity. Cultured cells were harvested for total RNA extraction using TRIzol Reagent (15596026, Invitrogen, Carlsbad, California, United States), and cDNA was synthesized using a Reverse Transcription Kit (R211-01, Vazyme, Nanjing, China). Subsequently, we performed on a LightCycler 96 instrument using an SYBR Green qPCR Kit (Q111, Vazyme, Nanjing, China). Gene expression was calculated using the 2^−ΔΔCT^ method with *β*-actin as a reference [21]. Specific primers for the target genes are listed in (Table 1).

### 2.2. Data Collection and Apoptosis Score

The clinical data and RNA-seq data of PRAD patients were obtained from TCGA database (https://portal.gdc.cancer.gov), and the FPKM format of TCGA-PRAD RNA-Seq data was converted to TPM and log2-fromat. A total of 495 PRAD and 52 paracancer control samples with progression-free interval (PFI) were included in this study. In addition, RNA-seq data and information of 131 patients with PRAD from the cBioPortal database (MSKCC, Cancer Cell 2010) were downloaded. The gene set of “HALLMARK_APOPTOSIS” was obtained from the MSigDB [22] (https://www.gsea-msigdb.org/gsea/msigdb/), and single sample gene set enrichment analysis (ssGSEA) was performed to calculate the apoptosis feature score for the patients using “GSVA” R package.

### 2.3. WGCNA

The “WGCNA” R package was employed to conduct WGCNA analysis for sectioning gene coexpression modules related to the apoptosis feature score [23]. First, the outlier samples were removed by hierarchical clustering, and a similarity matrix was generated using the Pearson method and transformed into an adjacency matrix for the gene-free scale network distribution. The correlation between connectivity (*K*) and *p*(*k*) reaching 0.85 was considered a scale-free topology criterion, and the pickSoftThreshold function was used to determine the optimal soft threshold (*β*). Finally, the adjacency matrix was calculated and denoted as *β*, and the gene expression profile was converted to a TOM matrix using dissTOM (method = “average”). Gene modules were sectioned using the cutreeDynamic function, and the apoptosis feature–related module was determined according to the Spearman correlation.

### 2.4. Differentially Expressed Genes (DEGs) and Function Enrichment Analysis

Under the screening threshold of |log_2_FC| > 1 and *p*.adj < 0.05, DEG analysis between the PRAD and paracancer control samples in the TCGA cohort was conducted with the “limma” R package. Then, the “clusterProfiler” R package was applied for the Gene Ontology (GO) and Kyoto Encyclopedia of Genes and Genomes (KEGG) enrichment analyses on these genes [24, 25]. The “clusterProfiler” R package was employed for significant pathway enrichment analysis based on the gene sets of HALLMARK pathway and GSEA; moreover, the HALLMARK gene set was also used in hallmark pathway activation with the “GSVA” R package [24].

### 2.5. Construction and Validation of Risk Model

Univariate Cox proportional hazard regression in the Kaplan–Meier (KM) “survival” R package was applied to screen significant prognostic factors (*p* < 0.05) [26], from which the hub genes were filtered by Lasso Cox regression analysis and stepwise regression and used to develop a RiskScore system with the “glmnet” R package: RiskScore = *Σβi* × Expi (*β* represents the regression coefficient of the gene; Exp represents the gene expression) [26]. For risk stratification, the RiskScore of patients was calculated based on the optimal cutoff point determined by the “survminer” R package [27].

### 2.6. Analysis of Immune Cell Infiltration

The CIBERSORT and ESTIMATE algorithms were used to evaluate immune infiltration in the TCGA cohort. The scores of 28 types of tumor-infiltrating lymphocytes (TILs) were calculated using the gene marker set from a previous study using the GSVA package [28]. The gene set of immune checkpoint genes from another published study was used to assess tumor malignancy [29]. Based on the somatic mutation profiles of TCGA and TCGA genomic mutation characteristics obtained from published research, tumor mutation burden (TMB) and mutation frequency of the patients were calculated using the “maftools” R package and the mafCompare function with Fisher's test [30].

### 2.7. Transfection Experiment

si-negative control (si-NC) and si-EPHB1 (#1 (sense strand (SS): 5⁣′- GUGGGAAACCAAAUAUAUAAU-3,⁣′ anti-sense strand (AS): 5⁣′- UAUAUAUUUGGUUUCCCACGG-3⁣′) and #2 (SS: 5⁣′-GAUGUUCAACAGAAGUGAAGA-3⁣′; AS: 5⁣′- UUCACUUCUGUUGAACAUCAC-3⁣′)) from Sangon Biotech (Shanghai, China) was used to silence EPHB1 in 22RV1 and PC-3 cells using the pLKO.1 vector (Zeye Biotech, Shanghai, China) with ampicillin and puromycin resistance. All constructs were confirmed by sequencing, and cell transfection was performed using Lipofectamine 3000 (L3000-001, Invitrogen, United States) [21]. To inhibit the activity of *GSK3B*, the specific inhibitor GSK3B Inhibitor XI (Compound 33, HY-112388, MedChemExpress, Monmouth Junction, New Jersey, United States) was used for intervention. The 22RV1 and PC-3 cells were inoculated in 6-well plates, and were treated by adding complete medium containing GSK3B Inhibitor XI (final concentration of 10 *μ*M) for 24 h once they grew attached to the edge of the plate. Such group was set up as the *GSK3B*-IN group; the cells of the control group were added with an equal volume of solvent (DMSO) and set up as the vector group. Cells were collected at the end of treatment for subsequent analysis.

### 2.8. Cell Viability, Invasion, and Proliferation Assay

A total of 4 × 10^3^ cells were seeded into 96-well plates for 2-h incubation, followed by adding 10 *μ*L of Cell Counting Kit-8 (CCK-8) (Dojindo, Kumamoto, Japan) to each well. The absorbance at 450 nm was measured using an enzyme-labeled instrument (iBIO-RADMark, Bio-Rad Laboratories) at 12, 24, and 36 h after cell culture based on the following formula: cell viability(%) = (*A*(CCK − 8) − *A*(control)) × 100%) [31]. Cell invasion activity was assessed using a transwell assay. The diluted matrix gel was added to the upper chamber of 24-well plates (Corning, Inc., Corning, New York, United States) containing 8-*μ*m pore inserts for 1-h incubation until a film was formed. After clearing excess fluid, 200 *μ*L of serum-free DMEM was added until the membrane became hydrated. Next, 5 × 10^4^ cell suspensions were seeded into the upper chamber, while the lower chamber was loaded with 800 *μ*L of DMEM containing 20% FBS. After 48 h of culture, 4% paraformaldehyde and 0.1% crystal violet (G1014-50ML, Servicebio, Wuhan, China) were used for cell fixation and staining, respectively, and cells were photographed under an inverted microscope (Leica, Wetzlar, Germany) [21, 32]. Finally, the BeyoClick EdU Kit (Beyotime, Hangzhou, China) was used to detect cell proliferation according to the manufacturer's specifications. Briefly, 1 × 10^4^ cells were cultured in the 6-well plates overnight, and preheated EdU solution (2x) at 37°C was then added to the 6-well plate in equal volumes for 2 h incubation. Next, the culture solution was removed, and 4% paraformaldehyde was added for cell fixation for 15 min. After washing the cells three times with PBS, PBS containing 0.3% Triton X-100 was added for 15-min incubation. After removing the washing solution, 1 mL of 1× Hoechst 33342 was added to stain cell nuclei. Fluorescence was detected using a fluorescence microscope (Leica, Germany) [33].

### 2.9. Co-Immunoprecipitation (Co-IP)

The cultured cells were harvested for the protein extraction using a RIPA buffer (Solarbio, Beijing, China) together with phosphatase inhibitors and a cocktail of protease (Solarbio, China), and the antibodies for Co-IP included anti-EPHB1 antibody (XGK1274, AmyJet Scientific Inc., Wuhan, China) and anti-GSK3B antibody (FNab03673, Feien Biotech, Wuhan, China). Next, 200 *μ*L of Pierce Protein A/G magnetic beads (Invitrogen, United States) was used to load the antibodies for 4 h, and 500 *μ*L of cell lysates was mixed with the antibody-crosslinked beads. The beads were washed five times with washing buffer (Cayman, Ann Arbor, Michigan, United States), 2 × sample loading buffer (Beyotime, China) was added, and the beads were incubated for 10 min. Finally, the lysates were separated using 10% sodium dodecyl sulfate–polyacrylamide gel electrophoresis (SDS-PAGE) for western blotting (see the following) [21].

### 2.10. Western Blotting

The protein samples were extracted from cultured cells and analyzed using a BCA standard protein kit (BCA01, Dingguo, Guangzhou, China). Then the proteins were separated by SDS-PAGE, electroblotted on PVDF membrane (Immobilon-P, Merck Millipore Burlington, Vermont, United States) and blocked with 5% nonfat milk for 1 h. Next, the primary rabbit antibodies of BCL2 (SAB4300339, Merck Millipore, United States), BAX (MBS840953, AmyJet Scientific Inc., China), Snail (ybs-1371R, Yanjin Biotech, Shanghai, China), E-CAD (FNab02618, Feien Biotech, China), N-CAD (K21283-JQK, Beijing Bio-Lab Technology, Beijing, China), SMAD3 (CAF50332, AmyJet Scientific Inc., China), p-SMAD3 (HZK-933951, Huzheng Biotech, Shanghai, China), p-GSK3B (69455-61-2, Ruiqi Biotech, Shanghai, China), and GAPDH (YT778-KFT, Beijing Bio-Lab Technology, China) were added for incubation at 4°C overnight. After washing with TBST, goat anti-rabbit IgG secondary antibody (ab205718, Abcam, Cambridge, United Kingdom) conjugated with horseradish peroxidase was used to incubate the protein for 2 h. Finally, ECL chemiluminescent liquid (PE0010, Solarbio) was used for protein detection using ImageJ software [34, 35].

### 2.11. Coculture of the Cells With Macrophages

A total of 5 × 10^5^ human monocytes (THP-1) obtained from ATCC were cultured in RPMI-1640 culture medium containing 10% FBS, 1% P/S, and 20 nM CSF-1 at 37°C in a 5% CO_2_ atmosphere. After 1 week of induction, monocyte-derived macrophages (MDMs) were obtained and further maintained in serum-free RPMI-1640 medium for 24 h to stabilize the M0 macrophage phenotype. For the coculture experiments, a total of 2 × 10^4^ 22RV1 or PC-3 cells with si-*EPHB1* and *GSK3B*-IN were seeded into the upper inserts of 24-well Transwell chambers (0.4 *μ*m pore size, Corning, Inc., United States), while M0 macrophages were plated in the lower chamber wells. The two cell types were cocultured for 48 h at 37°C to allow paracrine interaction without direct cell contact. After coculture, macrophages were harvested for immunofluorescence staining to assess phenotypic polarization.

### 2.12. Immunofluorescence Detection

After 48-h of incubation, 4% paraformaldehyde was applied for 30 min for cell fixation, and 0.5% Triton X-100 was added for another 2 min of incubation. Next, after blocking specific antibody binding with 1% bovine serum albumin for 1.5 h, anti-rabbit CD86 antibody (abs102644, Absin Bioscience Inc., Shanghai, China) and anti-rabbit CD206 antibody (MBS179821, AmyJet Scientific Inc., China) were added and incubated at 4°C overnight. Goat anti-rabbit IgG secondary antibody with DyLight 488 fluorescence label was used to incubate the cells for 2 h, and the nuclei were counterstained with DAPI. The cells were photographed using an upright fluorescent microscope [34].

### 2.13. Statistical Analysis

All statistical data were analyzed using the R software (Version 3.6.0) and GraphPad Prism software (Version 8.0.2). The Student's *t*-test was used to calculate the difference between two sets of continuous variables, and the two-way ANOVA was applied for the analysis on the results from CCK-8 assay. The KM survival analysis with log-rank test was conducted using the “survival” R package [27], and the “pROC” R package was used for the receiver operating characteristic (ROC) analysis with the area under curve (AUC). Three independent replications were performed for all experimental procedures. A *p* < 0.05 was of statistical significance (⁣^∗^*p* < 0.05, ⁣^∗∗^*p* < 0.01, ⁣^∗∗∗^*p* < 0,001, ⁣^∗∗∗∗^*p* < 0.0001) [27].

## 3. Results

### 3.1. Construction and Validation of a RiskScore Prognostic Model

**Figure 1:**
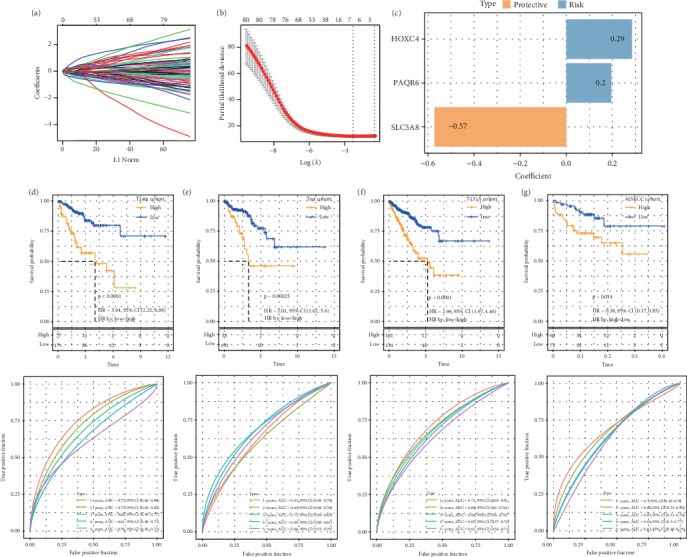
Construction and validation of model. (a) LASSO coefficient path plot. (b) LASSO regularization path plot. (c) Model gene and its coefficient. (d–g) KM survival analysis and ROC analysis in the training set, test set, TCGA cohort, and MSKCC cohort.

Based on the apoptosis gene set from the MSigDB database, we found that the apoptosis feature score was significantly lower in the tumor samples (*p* < 0.05, Figure S1A). WGCNA was used to identify the gene modules related to the apoptosis score. A soft threshold of *β* = 6 (Figure S2A,B) ensured a scale-free network for hierarchical clustering and generated 41 coexpression modules (Figure S1B). Notably, the red module was positively correlated with the apoptosis feature score (*p* = 1.10e − 64, Figure S1C,D), and its genes were mainly enriched in the focal adhesion, calcium signaling pathway, and cAMP signaling pathways in the KEGG analysis (Figure S1E) and were associated with the regulation of membrane potential, cell-substrate adhesion, muscle system process, urogenital system development, muscle contraction, and renal system development in the biological process (BP) term in the GO analysis (Figure S1F). We identified 348 overlapping genes between the DEGs (240 up-regulated DEGs and 759 down-regulated DEGs) and red module genes (Figure S3A–C). The training set in the TCGA-PRAD cohort was subjected to a chi-squared significant difference test at a ratio of 7:3. Three candidate genes were selected from 384 overlapping genes using the LASSO algorithm with stepwise regression (Figure 1) to formulate a RiskScore model = 0.285∗*HOXC*4 + 0.196∗*PAQR*6 + (−0.572∗*SLC*5*A*8) (Figure 1). KM survival analysis revealed that high-risk patients had poor outcomes and that the RiskScore model had an accurate classification ability (Figures 1, 1, 1, and 1), which supported the development of a nomogram with strong prediction performance and clinical value (Figure S4A–E).

### 3.2. Pathway Activation and Mutation Characteristic Analysis

**Figure 2:**
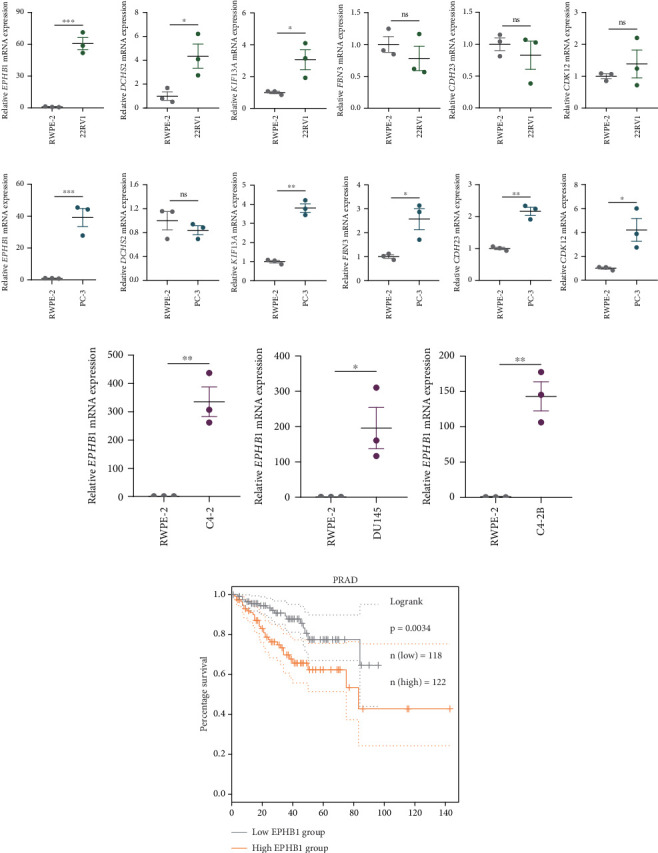
qPCR for the gene expression. (a, b) The expression of *EPHB1, DCHS2*, *KIF13A*, *FBN3*, *CDH23*, and *CDK12* in RWPE-2, 22RV1, and PC-3 cells. (c–e) The expression of *EPHB1* in RWPE-2, C4-2, DU145, and C4-2B cells. (f) KM survival analysis of *EPHB1* expression on PRAD prognosis (ns is *p* > 0.05, ⁣^∗^*p* < 0.05, ⁣^∗∗^*p* < 0.01, ⁣^∗∗∗^*p* < 0.001).

**Figure 3:**
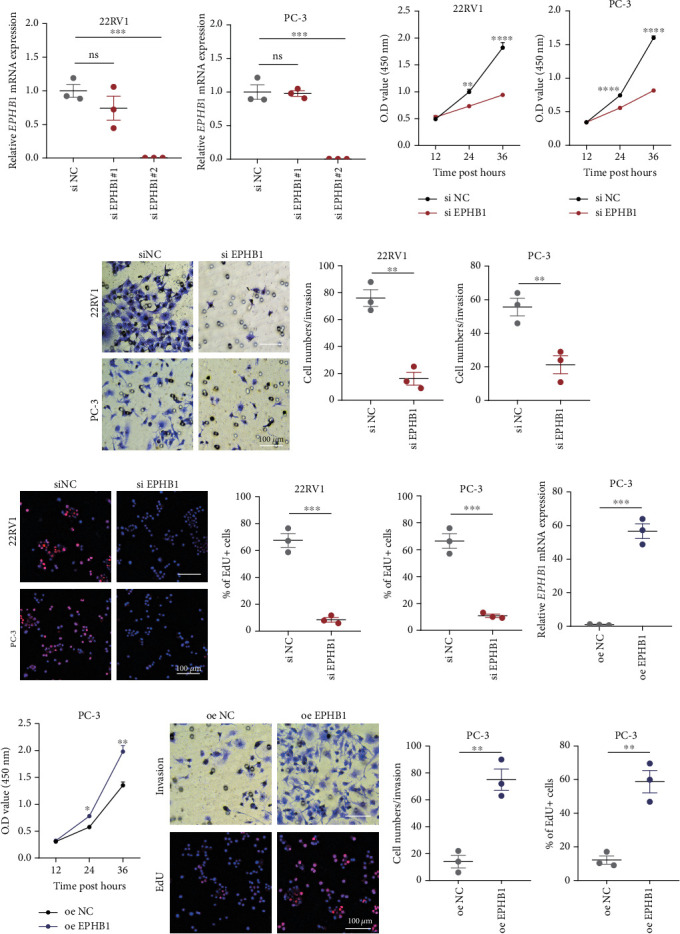
Cell viability, invasion, and proliferation test. (a, b) The expression of *EPHB1* in 22RV1 and PC-3 cells after *EPHB1* silencing. (c, d) CCK-8 for cell viability test after *EPHB1* silencing. (e, f) Trans-well for cell invasion test after *EPHB1* silencing. (g, h) Cell proliferation test after *EPHB1* silencing. (i, j) The cell viability test after overexpression of *EPHB1*. (k, l) Cell invasion test after overexpression of *EPHB1*. (m) Cell proliferation test after overexpression of *EPHB1* (⁣^∗^*p* < 0.05, ⁣^∗∗^*p* < 0.01, ⁣^∗∗∗^*p* < 0.001, ⁣^∗∗∗∗^*p* < 0.0001).

GSEA revealed that high-risk patients had significantly enriched DNA replication initiation, meiotic cell cycle phase transition, and DNA replication checkpoint signaling pathway activation (Figure S5A). The hallmark analysis showed that proliferation-related pathways, including E2Ftargets and the G2Mcheckpoint, were significantly activated in the high-risk group (Figure S5B), which also showed a significantly higher rate of nonsilent mutation, number of segments and fraction altered, proliferation, and homologous recombination defect score (*p* < 0.05, Figure S5C) and higher mutation frequencies of *EPHB1*, *DCHS2*, *KIF13A*, *FBN3*, *CDH23*, and *CDK12* (Figure S5D). We observed a trend of upregulation of multiple genes (especially *EPHB1* and *KIF13A*) in two PRAD cell lines (22RV1 and PC-3) (*p* < 0.05, Figure 2), and *EPHB1*, with the highest mutation frequency, was also significantly upregulated in C4-2, DU145, and C4-2B tumor cells (*p* < 0.05, Figures 2, 2, and 2). Based on median *EPHB1*, KM survival analysis demonstrated that patients with high *EPHB1* had poor clinical outcomes (Figure 2). After silencing *EPHB1* in the 22RV1 and PC-3 cells, si-EPHB1#2 exhibited a higher silencing efficiency (Figure 3). Meanwhile, cell viability (Figure 3) and cell invasion activity (Figure 3) were significantly reduced after 24 and 36 h of culture. In addition, the EdU method revealed lower cell proliferation in the si-EPHB1 group (Figure 3), and *EPHB1* was significantly overexpressed in the oeEPHB1 group with high overexpression efficiency of oeEPHB1 (Figure 3). Overexpression of *EPHB1* increased tumor cell viability (Figure 3), invasion, and proliferation (Figures 3, 3, and 3). These results indicate that high expression of *EPHB1* affected the proliferation, invasion, and survival of tumor cells, suggesting its vital role in tumor progression in high-risk PRAD patients.

### 3.3. The EPHB1-GSK3B-SMAD3 Pathway Affected the Cell Viability and Apoptosis

**Figure 4:**
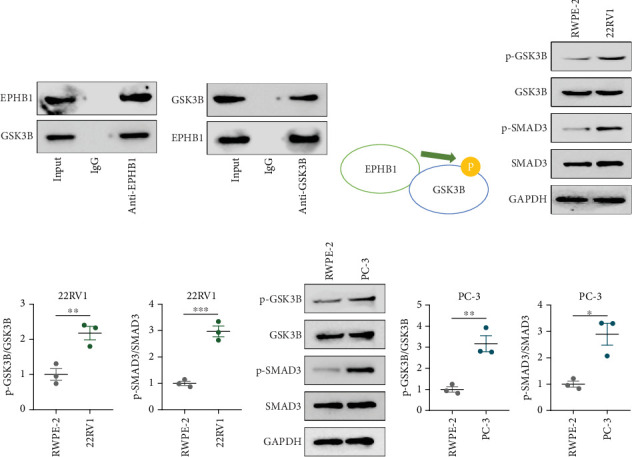
Phosphorylation function verification of EPHB1. (a, b) Co-IP for the interaction between *EPHB1* and *GSK3B* protein. (c) Phosphorylation model of *EPHB1* and *GSK3B* protein. (d, e) The phosphorylation levels of *GSK3B* and *SMAD3* protein in the 22RV1 cells. (f, g) The phosphorylation levels of *GSK3B* and *SMAD3* protein in the PC-3 cells (⁣^∗^*p* < 0.05, ⁣^∗∗^*p* < 0.01).

**Figure 5:**
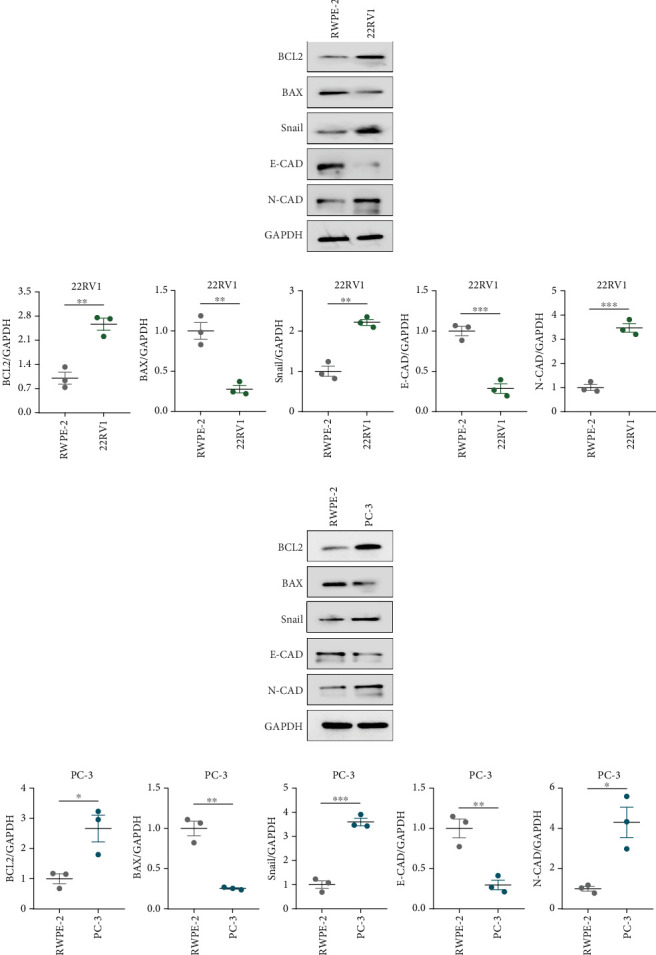
Detection of apoptosis-related protein expression. (a, b) Western blot of BCL2, BAX, Snail, E-CAD, N-CAD, and GAPDH protein expression in 22RV1 cells. (c, d) Western blot of BCL2, BAX, Snail, E-CAD, N-CAD, and GAPDH protein expression in PC-3 cells (⁣^∗^*p* < 0.05, ⁣^∗∗^*p* < 0.01, ⁣^∗∗∗^*p* < 0.001).

A Co-IP experiment was performed to detect the relationship between EPHB1 and GSK3B protein, which has been reported to be involved in apoptosis, migration, and invasion in prostate cancer [36], and the results confirmed the interaction between EPHB1 and GSK3B protein (Figure 4a,b). A previous study also showed that upregulated secreted frizzled-related protein 1 (sFRP1) in the prostatic tumor stroma [37] could restore the activity of glycogen synthase kinase 3*β* (GSK3*β*), whereas inhibition of GSK3*β* abolishes the regulation of sFRP1 on SMAD3 signaling and the aggressive phenotype of gastric cancer [38]. We found that GSK3B and the proliferative regulatory protein SMAD3 were phosphorylated substrates of the EPHB1 protein (Figure 4c). Here, western blot analysis showed that the expression of p-GSK3B and p-SMAD3 in 22RV1 cells (Figure 4d,e) and PC-3 tumor cells (Figure 4f,g) was significantly upregulated compared to that in RWPE-2 cells, suggesting that the EPHB1-GSK3B-SMAD3 signaling pathway was activated during PRAD development. The expression of the antiapoptotic protein BCL2 and proinvasion-related proteins *Snail* and *N-CAD* was upregulated in 22RV1 and PC-3 tumor cells, while those of the proapoptotic protein BAX and invasion-related protein E-CAD were downregulated (Figures 5a, 5b, 5c, and 5d).

### 3.4. GSK3B Downregulation Inhibited Cell Viability and Promoted Expressions of Proapoptotic Proteins

**Figure 6:**
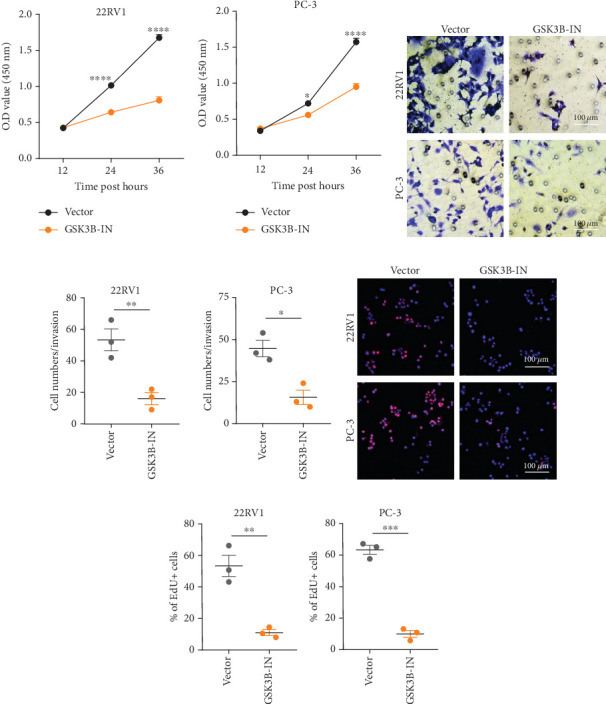
Viability, invasion, and proliferation assays on PRAD cells after GSK3B inhibition. (a, b) CCK-8 for assessing the effect on the proliferative capacity of PRAD cell lines after inhibition of *GSK3B*. (c, d) Trans-well for assessing the effect on the invasive capacity of PRAD cell lines after inhibition of *GSK3B*. (e, f) EdU assay to be used to assess the effect on the proliferation level of 22RV1 and PC-3 cells after inhibition of *GSK3B* (⁣^∗^*p* < 0.05, ⁣^∗∗^*p* < 0.01, ⁣^∗∗∗^*p* < 0.001, ⁣^∗∗∗∗^*p* < 0.0001).

**Figure 7:**
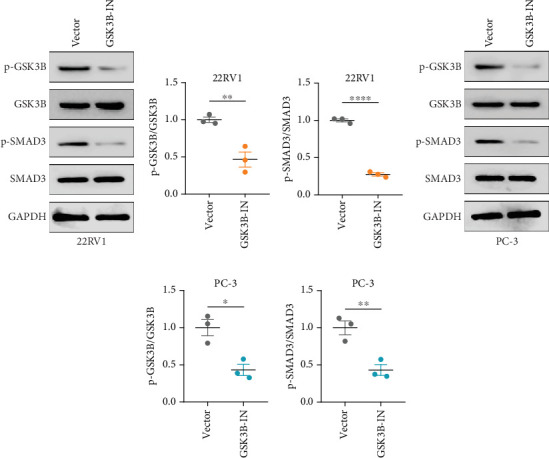
Phosphorylation function verification of GSK3B inhibition. (a, b) Western blot of p-GSK3B, GSK3B, p-SMAD3, SMAD3, and GAPDH protein expression after *GSK3B* inhibition in 22RV1 cells. (c, d) Western blot of p-GSK3B, GSK3B, p-SMAD3, SMAD3, and GAPDH protein expression after *GSK3B* inhibition in PC-3 cells (⁣^∗^*p* < 0.05, ⁣^∗∗^*p* < 0.01, ⁣^∗∗∗∗^*p* < 0.0001).

**Figure 8:**
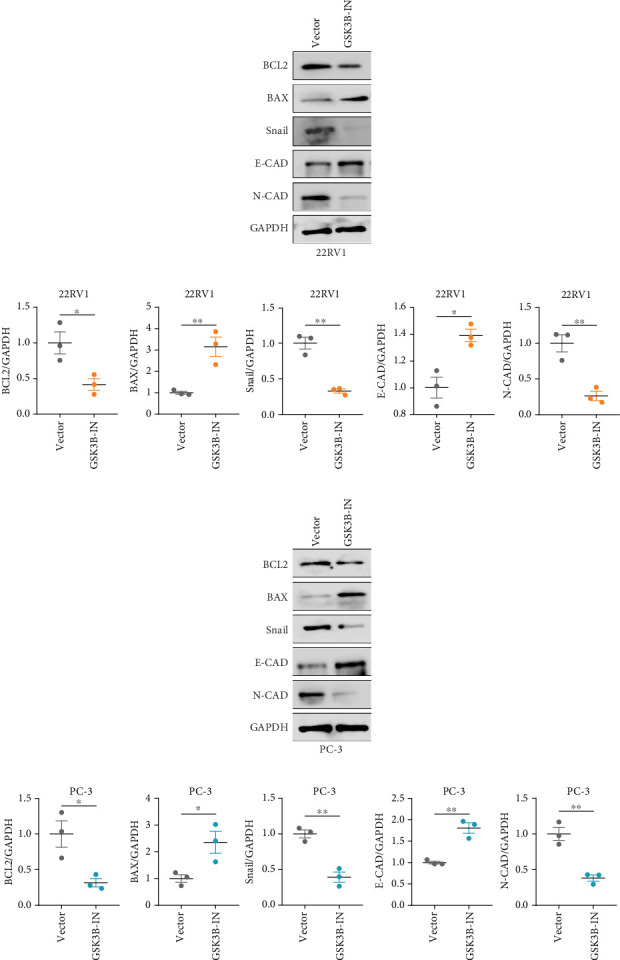
Detection of apoptosis and invasion-related protein expression after GSK3B inhibition. (a, b) Western blot of BCL2, BAX, Snail, E-CAD, N-CAD, and GAPDH protein expression in 22RV1 cells after *GSK3B* inhibition in 22RV1 cells. (c, d) Western blot of BCL2, BAX, Snail, E-CAD, N-CAD, and GAPDH protein expression in 22RV1 cells after *GSK3B* inhibition in PC-3 cells (⁣^∗^*p* < 0.05, ⁣^∗∗^*p* < 0.01).

We used inhibitor for *in-vitro GSK3B* intervention (GSK3B-IN) assay; the viability of 22RV1 and PC-3 cells was significantly inhibited after 24 and 36 h of culture in the GSK3B-IN group (*p* < 0.05, Figure 6), and the cell invasion and proliferation activities were also inhibited (Figures 6, 6, 6, and 6). Meanwhile, we also examined the effect of *GSK3B* inhibition on p-GSK3B and p-SMAD3 expression. *GSK3B* inhibition significantly suppressed the levels of p-SMAD3, BCL2, Snail, and N-CAD (Figures 7, 7, 7, and 7) but promoted the expression of BAX and E-CAD in 22RV1 and PC-3 cells after GSK3B-IN interference (Figures 8, 8, 8, and 8).

### 3.5. EPHB1 and GSK3B Expression Promoted Macrophage M2 Polarization

**Figure 9:**
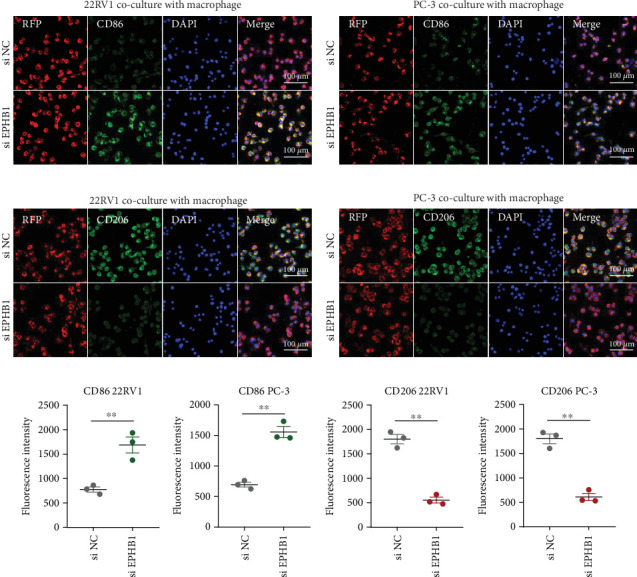
Coculture assay for testing the effect of EPHB1 silencing on macrophage polarization. (a–d) The coculture of 22RV1/PC-3 and macrophage after *EPHB1* silencing. (e, f) The CD86 and CD206 fluorescence intensity detection (⁣^∗∗^*p* < 0.01).

**Figure 10:**
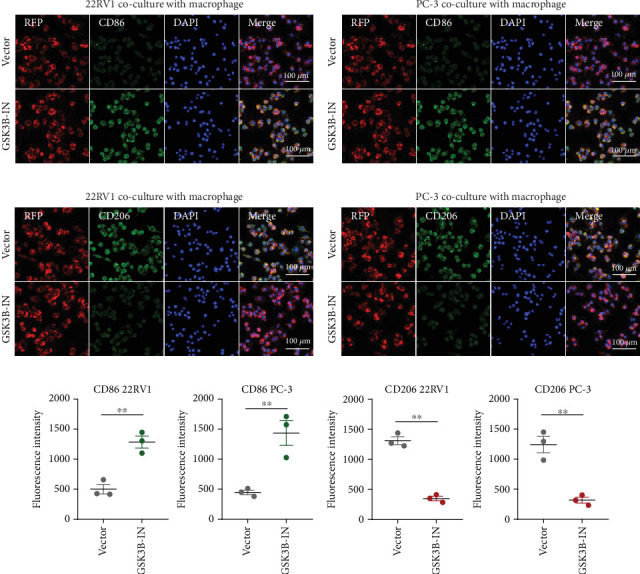
Coculture assay for testing the effect of GSK3B inhibition on macrophage polarization. (a–d) The coculture of 22RV1/PC-3 and macrophage after *GSK3B* inhibition. (e, f) The CD86 and CD206 fluorescence intensity detection after *GSK3B* inhibition (⁣^∗∗^*p* < 0.01).

After the coculture of 22RV1/PC-3 cells and macrophages, we found that 22RV1 cells reduced the number of macrophages expressing the CD86 marker, whereas 22RV1 cells with si-EPHB1 upregulated the percentage of CD86+ macrophages (Figure 9). Consistently, the cells of PC-3 cocultured with macrophages showed similar results (Figure 9). In addition, we found that 22RV1/PC-3 cells cocultured with macrophages upregulated the percentage of CD206+ macrophages and that tumor cells with si-EPHB1 inhibited the percentage of CD206+ macrophages (Figure 9). These findings suggest that the tumor cells induced macrophage polarization to the M2 type (CD206 marker), while si-*EPHB1* silencing in tumor cells mediated M1 macrophage polarization (CD86 marker) (Figure 9). At the same time, consistent results were observed in the *GSK3B*-IN groups in that *GSK3B* downregulation mediated macrophage M1 polarization (CD86 marker) in tumor cells (Figures 10, 10, 10, 10, 10, and 10).

### 3.6. Immune Infiltration Features in the High- and Low-Risk Groups

A comparison of the differences in tumor microenvironment (TME) revealed that the immune, stromal, and ESTIMATE scores were significantly higher in the low-risk group (*p* < 0.05, Figure S6A), indicating fewer stromal and immune components in the high-risk group. The CIBERSORT algorithm revealed significantly low levels of CD4 memory resting T cells and high levels of T cell regulatory cells (Tregs), activated NK cells, and M2 macrophage infiltration in the high-risk group (*p* < 0.05, Figure S6B). In addition, ssGSEA data demonstrated that the enrichment scores of most immune cells, such as activated B cells, effector memory CD8 T cells, memory B cells, regulatory T cells, and neutrophils, were significantly higher in the low-risk group (Figure S6C). The immune checkpoint genes *BTLA*, *CD27*, *CD28*, *CTLA4*, *VTCN1*, *SIRPA*, and *LGALS9* were highly expressed in the low-risk group (*p* < 0.05; Figure S6D), indicating that low-risk patients may benefit from immune checkpoint blocking therapy. TMB analysis showed that high-risk patients had significantly higher TMB scores (*p* < 0.05; Figure S6E), indicating that these patients were more sensitive to immunotherapy.

## 4. Discussion

PRAD is a common male malignancy with unfavorable treatment outcomes, especially in advanced patients who progress to castration-resistant PRAD (resistant to chemotherapy, radiotherapy, and ADT treatment) [39]. Currently, molecular markers have served as promising risk stratification tools for the prognostic and immune treatment response assessment [40, 41]. In this study, a prognostic risk score model was developed for PRAD. Specifically, we examined the function of the highly mutant gene *EPHB1* and observed that overexpression of *EPHB1* in PRAD cells upregulated the expression of antiapoptotic and invasion markers *BCL2, Snail*, and *N-CAD* proteins and promoted cell viability, proliferation, and invasion. Meanwhile, *EPHB1* protein interaction with *GSK3B* protein enhanced the phosphorylation levels of *GSK3B* and *SMAD3*, while the knockdown of *GSK3B* reduced the phosphorylation of *SMAD3*. Based on these findings, it can be concluded that *EPHB1* played a crucial role in activating the EPHB1-GSK3B-SMAD3 pathway to support PRAD progression. To the best of our knowledge, this may be a novel cancer-promoting signaling pathway in PRAD that has not been previously reported. Our findings provide a basic reference for understanding the mechanism of PRAD progression.

In our model, *SLC5A8* upregulation reduced patients' risk, but the upregulation of *HOXC4* and *PAQR6* increased patients' risk, indicating their potential as molecular targets for gene therapy. *SLC5A8* is a tumor-suppressive transporter in cervical cancer (CC), and its overexpression suppresses proliferation and induces apoptosis by inhibiting Wnt signaling [42] and arresting HeLa cells at the G1 phase [43]. Overexpression of *SLC5A8* mediates apoptosis induction for the antitumor response in liver cancer [44]. The findings of Luo et al. are similar to our findings. They also found that the transcription factor Homeobox C4 (*HOXC4*) is a clinical biomarker for aggressive PRAD and that abnormal upregulation of *HOXC4* aggravates PRAD progression [45]. *PAQR6* is significantly upregulated in PRAD tissues and is closely associated with a higher pathological stage of the tumor, ratio of free-prostate-specific antigen/total-PSA, Gleason score, and shorter OS [46]. These findings support our findings that changes in the RiskScore were consistent with gene functions, and these model genes can be considered as potential target genes for PRAD therapy.

*EPHB1* was the gene with the highest mutation frequency in high-risk patients, and high *EPHB1* in the PRAD cells was closely associated with poor prognosis. We also observed that *EPHB1* mediated cancer-promoting traits underlying PRAD progression. *EPHB1* is a member of the receptor tyrosine kinases that mediate the Eph receptor and ligand signaling transduction to regulate cell migration, differentiation, adherence, apoptosis, and proliferation in cancer [47]. For example, *SMAD2* activated by TGF-*β* signaling upregulates the expression of *EPHB1*, which further promotes epithelial–mesenchymal transition (EMT) by elevating *CDH2* expression in lung cancer [48]. *GSK3B* encodes a serine–threonine kinase involved in stress and apoptotic pathways. Xu et al. showed that *GSK3B*, downregulated by miR-132-3p, enhances etoposide-induced cell apoptosis in breast cancer [49] and that upregulated *SMAD3* promotes the expression of androgen receptor (AR), resulting in the development of CRPC [50]. To date, only a few studies have focused on the expression of *EPHB1*, *GSK3B*, and *SMAD3* in PRAD. Our study demonstrated that downregulation of *EPHB1* and *GSK3B* suppressed the phosphorylation of *GSK3B* protein and *SMAD3* protein, respectively, and that *EPHB1* played a crucial role in activating EPHB1-GSK3B-SMAD3 signaling and affecting PRAD progression.

As crucial components of the TME, tumor-infiltrating immune cells participate in PRAD progression and affect tumor treatment response [51]. According to antitumor immunity, PRAD is considered as “cold tumor” with tumor suppression defects, low immunological infiltration, and low antigen activation in a suppressive TME [52], whereas some specific TILs may accelerate the development [53]. ESTIMATE analysis revealed that high-risk PRAD patients with lower immune and stromal scores lacked immune-active components; however, Tregs, NK cell activation, and macrophage M2 infiltration were significantly higher in the high-risk group. High levels of Tregs in biopsy samples of PRAD have been observed to block autoreactive T cells for antitumor responses [54]. M2 macrophages normally have an immunosuppressive function that inhibits antitumor T cell activity and stimulates tumor angiogenesis [55]. The current study found that the knockdown of *EPHB1* or inhibition of *GSK3B* promoted the fluorescence intensity of CD86 but reduced that of CD206 in PRAD cells cocultured with macrophages, indicating that *EPHB1* and *GSK3B* were key risk factors for promoting PRAD progression. In addition, high NK cell activity could delay the castration-resistant progression of PRAD [56]; some reports have also shown its protumorigenic traits in a highly immunosuppressive TME because of functional plasticity [57]. Overall, Tregs and M2 macrophages contributed to the immunosuppressive TME in PRAD.

In addition, we also found that high-risk PRAD patients were associated with activated proliferation pathways, including the cell cycle, DNA duplication, and meiosis, which may be explained by the effect of EPHB1-GSK3B-SMAD3 signaling pathway activation on promoting tumor cell viability, invasion, and proliferative activity, macrophage M2 polarization, and inhibition of the expression of antiapoptotic proteins. Overall, this study reports a novel EPHB1-GSK3B-SMAD3 pathway and emphasizes its importance in PRAD progression. However, this study has some limitations. First, this study was mainly based on public databases for bioinformatics analysis and functional validation at the cellular level, and protein level expression validation of key molecules such as *EPHB1*, *GSK3B*, and *p-SMAD3* has not yet been performed by clinical PRAD tissue samples, which lacks direct evidence support at the clinical sample level. Therefore, we will further collect primary and metastatic tissues from patients to assess the expression and localization of *EPHB1* and its related pathway proteins by immunohistochemistry and immunofluorescence, in order to further validate their expression patterns and relevance in the clinicopathological state. In addition, although we found that *EPHB1* interacts with *GSK3B* and that *GSK3B* knockdown affects the phosphorylation status of *SMAD3*, no direct evidence has been provided to confirm *SMAD3* as a direct substrate of *GSK3B*. In the future, in vitro kinase assays, mutant construction, and mass spectrometry identification will be used to further clarify the direct regulatory relationship in the EPHB1-GSK3B-SMAD3 pathway. Finally, immune microenvironment regulation has only been initially observed through the coculture system of macrophages and PRAD cells with M1/M2 polarization, and a comprehensive analysis of immune cell profiles is still lacking, and the involvement of key inflammatory factors or immune killer cells has not been assessed. Single-cell RNA sequencing and spatial transcriptome technology will be introduced in the follow-up, combined with tumor tissue immune infiltration mapping, to systematically assess the remodeling effect of the *EPHB1* pathway on the tumor immune microenvironment and to further clarify its effect on immune cells.

## 5. Conclusion

This study constructed a RiskScore and nomogram model with high classification efficiency and prediction accuracy based on apoptotic features in PRAD. *EPHB1* is the gene with the highest mutation frequency and is highly expressed in high-risk PRAD patients, whereas the knockdown of *EPHB1* inhibited cell viability, proliferation, invasion, and antiapoptotic activity. Meanwhile, *EPHB1* protein interacted with GSK3B and further affected the phosphorylation of SMAD3; notably, *EPHB1* and *GSK3B* functioned crucially in macrophage M2 polarization. In conclusion, this study emphasizes the significance of EPHB1-GSK3B-SMAD3 signaling in PRAD progression and provides a new theoretical basis and experimental support for future studies on the molecular mechanism of PRAD and the development of clinical treatment strategies for cancer.

## Figures and Tables

**Table 1:** The specific primer for qPCR.

**Gene**	**Forward (5**⁣′**-3**⁣′**)**	**Reversed (5**⁣′**-3**⁣′**)**
EPHB1	GACCAAGGTGAACTCGGTTCC	GGAGGAGGGGCTCTGAGGG
DCHS2	TGTATCACCCTGTTTTCTCACTCATG	GCTCAAATTCATCTATCCCTTCAAT
KIF13A	TGCCTAAGAATGAGCTTGCAAA	AGAATGGCAGCACGTGGCT
FBN3	GCATGGCAGGTGGCCAAG	GACAGCGAGATGGGTCTGGTG
CDH23	GACGCTGACCGCTGCCGA	TGCTGCTCACACCCACAAGG
CDK12	GAGAAGACCAGGAAAGAACGGG	AGATCGTGAGGGACTAAGGTCATAA
BCL2	CAACCGGGAGATGTCGCC	TCGCCGGCTCCACAGCCT
Snail	CTGAGTGCCCCACTTCTGGC	CACGCAGACAGGCCAGCT
N-CAD	TATGTGTACATAATGTTTTATTGGCATAGT	TCTAACTACAGCTCAACAATTGAATCA
BAX	ATGCGTTTTCCTTACGTGTCTGA	AGAGCTAGGGTCAGAGGGTCATC
E-CAD	GCTCACTGCAGCCTTGTCC	CAAGATGGGAGGATCACTTGAGC
*β*-Actin	ATCATGTTTGAGACCTTCAACACCC	CAGCCAGGTCCAGACGCA

## Data Availability

The datasets generated and/or analyzed during the current study are available at MSigDB (https://www.gsea-msigdb.org/gsea/msigdb/).

## Nomenclature

PRAD: prostate adenocarcinoma
WGCNA: weighted gene coexpression network analysis
DEGs: differentially expressed genes
RT: radiation therapy
ADT: androgen deprivation therapy
TCGA: The Cancer Genome Atlas
MSigDB: Molecular Signatures Database
GSEA: gene set enrichment analysis
TILs: tumor-infiltrating lymphocytes
TMB: tumor mutation burden
KM: Kaplan–Meier
ROC: receiver operating characteristic curve
AUC: area under curve

## Supporting Information

Supporting Information 1 Figure S1. WGCNA for the apoptosis feature–related module. (A) The difference in apoptosis score between the tumor and paracarcinoma samples. (B) The cluster of tree diagrams of WGCNA analysis. (C) Heatmap of correlation. (D) Scatter plot of the relationship between module membership and gene significance. (E) Bubble plot of KEGG channel enrichment analysis. (F) Bubble plot of biological process enrichment analysis.

Supporting Information 2 Figure S2. WGCNA for the outlier and soft threshold processing. (A) Sample clustering to detect outliers and soft threshold. (B) The gene numbers in each module.

Supporting Information 3 Figure S3. Screening of apoptosis characteristic genes. (A) The volcano plot of DEGs between tumor and paracarcinoma samples. (B) The heatmap plot of DEGs expression in different groups. (C) Venn plot for the overlapping genes.

Supporting Information 4 Figure S4. Identifying significant independent factors for nomogram model. (A, B) Univariate and multivariate Cox regression analysis of clinical features. (C) A nomogram model. (D) Calibration curve of nomogram model. (E) Decision curve of nomogram model.

Supporting Information 5 Figure S5. Pathway activation difference in different risk groups. (A) GSEA enrichment analysis in high-risk group. (B) The hallmark pathway activation difference in high and low risk groups. (C) Genomic characteristic difference in the high and low risk groups. (D) Differences in genetic mutations between high-risk and low-risk groups. (∗*p* < 0.05, ∗∗*p* < 0.01, ∗∗∗*p* < 0,001, ∗∗∗∗*p* < 0.0001).

Supporting Information 6 Figure S6. Immune infiltration analysis of different risk groups. (A) ESTIMATE analysis in the TCGA cohort. (B) Immune infiltration difference of 28 immune cells in different risk groups. (C) ssGSEA score difference in different risk groups. (D) THE immune checkpoint gene expression difference. (E) The tumor mutation burden difference analysis. (∗*p* < 0.05, ∗∗*p* < 0.01, ∗∗∗*p* < 0,001, ∗∗∗∗*p* < 0.0001).
