# Supplementary figures and images for: EPHB1 Protein Promoted the Progression of Prostate Adenocarcinoma Through Phosphorylating GSK3B and Activating EPHB1-GSK3B-SMAD3 Pathway
Source: Hum Mutat. 2025 Jun 12;2025:4961883. doi: 10.1155/humu/4961883 (PMC12178783; doi:10.1155/humu/4961883)

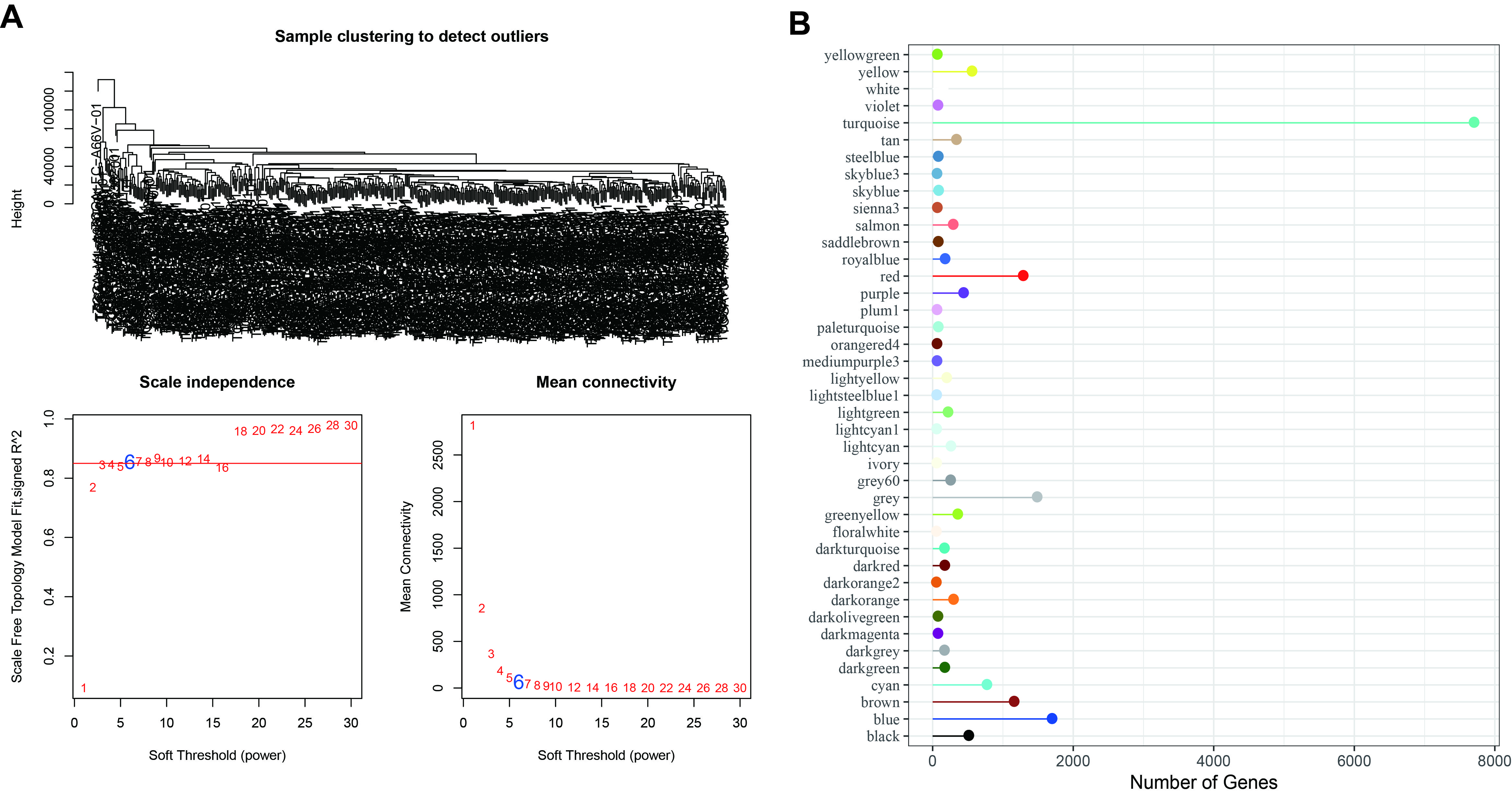

Supplement: Supporting Information 2 — Figure S2. WGCNA for the outlier and soft threshold processing. (A) Sample clustering to detect outliers and soft threshold. (B) The gene numbers in each module. [file 4961883.f2.jpg]

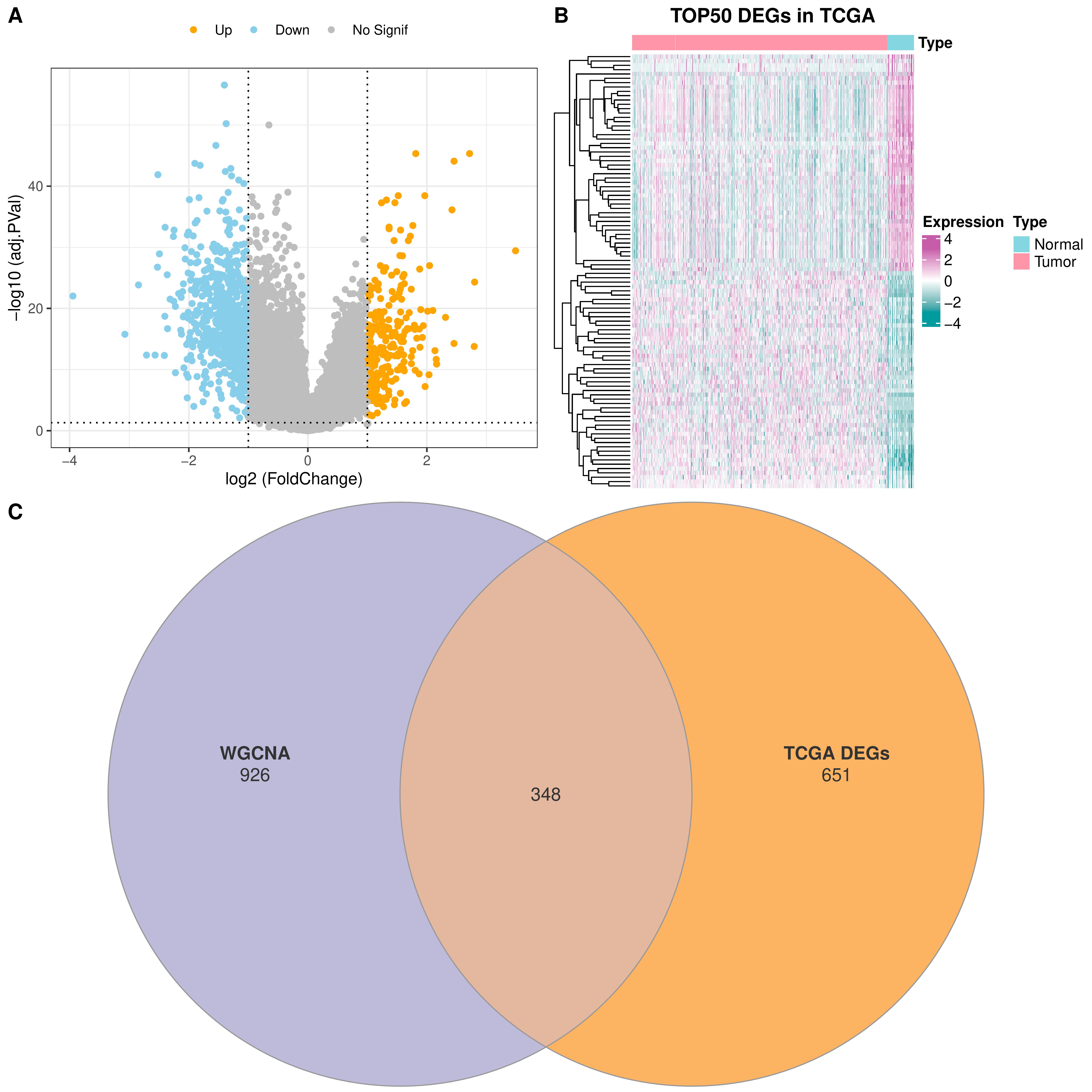

Supplement: Supporting Information 3 — Figure S3. Screening of apoptosis characteristic genes. (A) The volcano plot of DEGs between tumor and paracarcinoma samples. (B) The heatmap plot of DEGs expression in different groups. (C) Venn plot for the overlapping genes. [file 4961883.f3.jpg]

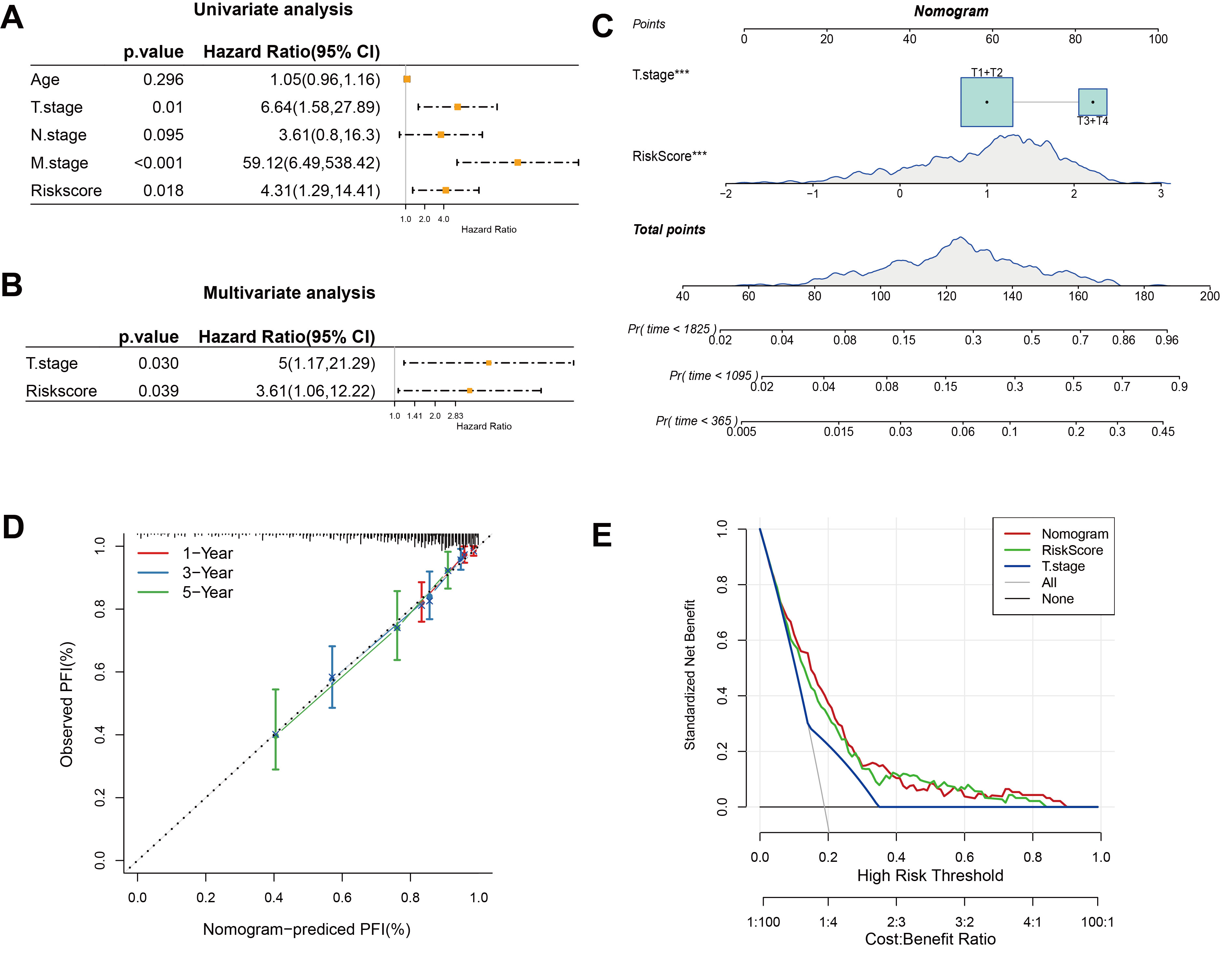

Supplement: Supporting Information 4 — Figure S4. Identifying significant independent factors for nomogram model. (A, B) Univariate and multivariate Cox regression analysis of clinical features. (C) A nomogram model. (D) Calibration curve of nomogram model. (E) Decision curve of nomogram model. [file 4961883.f4.jpg]

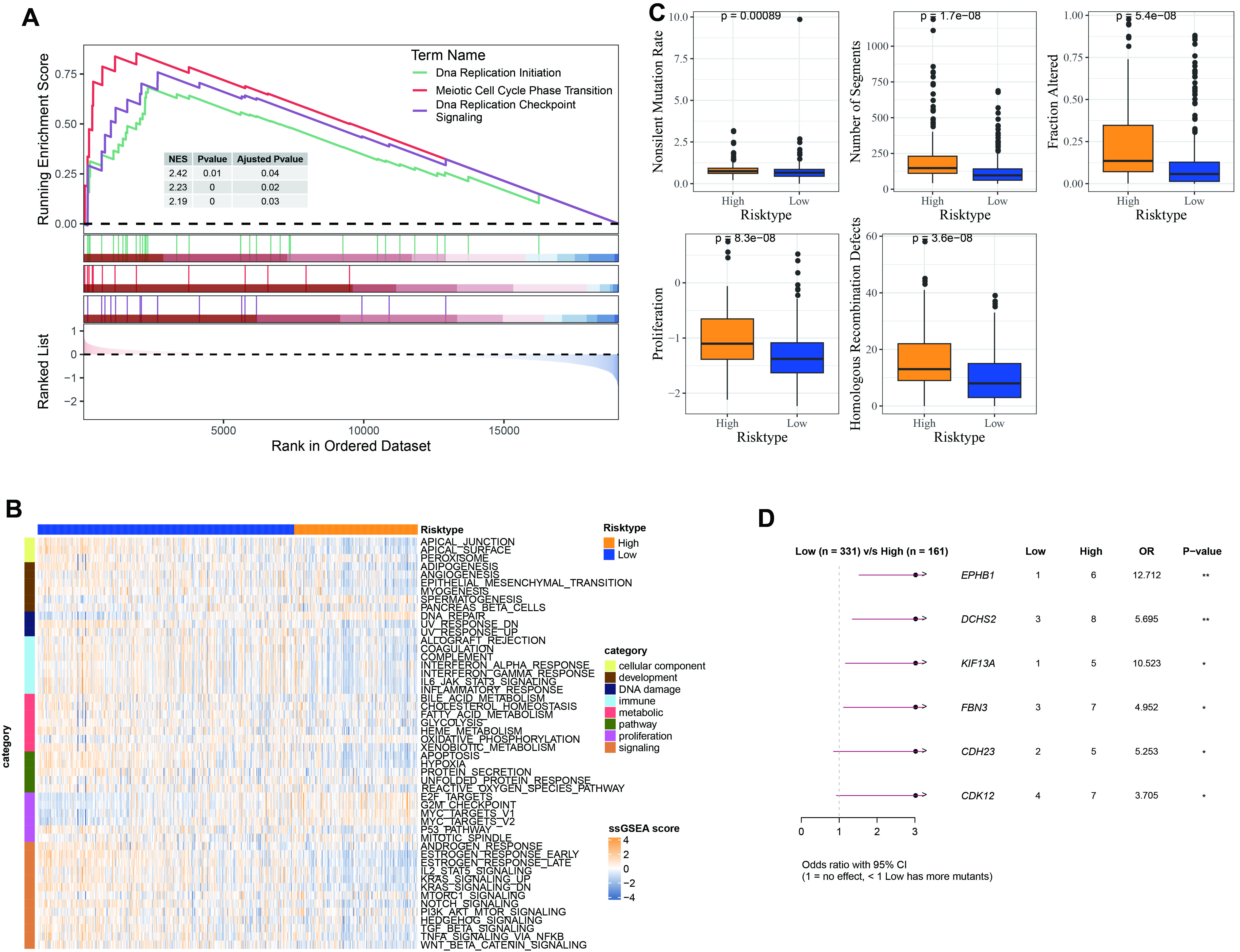

Supplement: Supporting Information 5 — Figure S5. Pathway activation difference in different risk groups. (A) GSEA enrichment analysis in high-risk group. (B) The hallmark pathway activation difference in high and low risk groups. (C) Genomic characteristic difference in the high and low risk groups. (D) Differences in genetic mutations between high-risk and low-risk groups. (∗p < 0.05, ∗∗p < 0.01, ∗∗∗p < 0,001, ∗∗∗∗p < 0.0001). [file 4961883.f5.jpg]

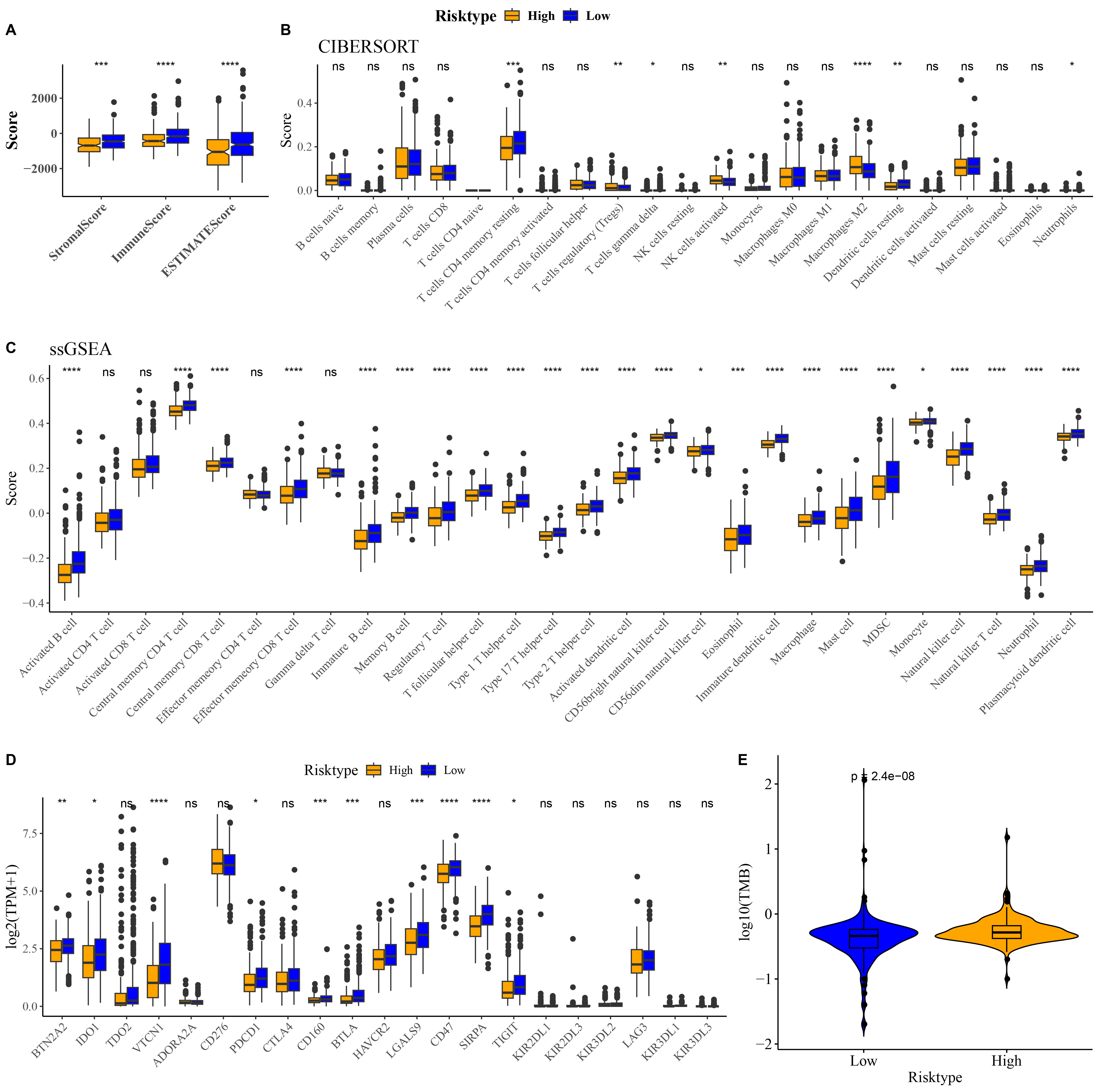

Supplement: Supporting Information 6 — Figure S6. Immune infiltration analysis of different risk groups. (A) ESTIMATE analysis in the TCGA cohort. (B) Immune infiltration difference of 28 immune cells in different risk groups. (C) ssGSEA score difference in different risk groups. (D) THE immune checkpoint gene expression difference. (E) The tumor mutation burden difference analysis. (∗p < 0.05, ∗∗p < 0.01, ∗∗∗p < 0,001, ∗∗∗∗p < 0.0001). [file 4961883.f6.jpg]
